# Increased human occupation and agricultural development accelerates the population contraction of an estuarine delphinid

**DOI:** 10.1038/srep35713

**Published:** 2016-10-19

**Authors:** Wenzhi Lin, Leszek Karczmarski, Jia Xia, Xiyang Zhang, Xinjian Yu, Yuping Wu

**Affiliations:** 1 South China Sea Bio-Resource Exploitation and Utilization Collaborative Innovation Center; Zhuhai Key Laboratory of Marine Bioresources and Environment, Guangdong Provincial Key Laboratory of Marine Resources and Coastal Engineering, School of Marine Sciences, Sun Yat-Sen University, Guangzhou 510275, PR China; 2 The Swire Institute of Marine Science and School of Biological Sciences, The University of Hong Kong, Cape d’Aguilar, Shek O, Hong Kong

## Abstract

Over the past few thousand years, human development and population expansion in southern China have led to local extirpation and population contraction of many terrestrial animals. At what extent this early human-induced environmental change has also affected coastal marine species remains poorly known. We investigated the demographic history of the Indo-Pacific humpback dolphin (*Sousa chinensis*) in the Pearl River Delta (PRD); an obligatory inshore species known for its susceptibility to anthropogenic impacts in one of China’s most developed coastal regions. Although the deltaic evolution of PRD has been influenced by climate since the Holocene, ~74% reduction of the dolphin’s effective population size occurred within the last 2000 years, consistent with ~61% habitat contraction during this period. This considerable and recent population contraction may have been due to land use practices and deforestation in the upper/middle Pearl River region, all leading to increasing sedimentation rate in the estuarine area. As anthropogenic impacts within the drainage of Pearl River affected a vast area, coastal dolphins and large terrestrial mammals in southern China may share a similar demographic history, whilst the demographic and biogeographic history of the PRD humpback dolphins may be symptomatic of similar processes that this species may have undergone elsewhere in the region.

Demographic history of populations is a product of dynamic processes where populations respond to environmental change, which in turn impacts the genetic diversity of individual populations, inter-population gene flow or connectivity, geographic distribution, and in time the evolution of species^1^. Drastic demographic expansions or declines of a species have been associated with climatic changes, geographic events, or human activities, which alter the food supply, size and patchiness of a habitat, or directly remove individuals^2^,^3^,^4^. A severe population contraction, known as bottleneck, increases the likelihood of stochastic events due to the increased drift in small populations, which could compromise the survivorship of species. Thus, identifying the major forces that determine the population demographic history is vital to understanding contemporary population processes and improving present-day environmental management.

The current rate of extinction of species is over 1,000 times faster than during the pre-human era^5^. According to the International Union for the Conservation of Nature (IUCN) Red List of Threatened Species (www.iucnredlist.org, date accessed: Jun 2016), over 20% of the 59,033 listed animal species are currently on the verge of extinction (subtotal of threatened *spp.*). This number may be an underestimate as the original population sizes of the majority of extant species and populations are unknown. It has been long recognized that demographic change leads to the variation in allelic frequency spectrum, such as an excess of heterozygosity^6^ or loss of rare alleles during a bottleneck^7^. Although genetic signatures of recent bottlenecks are generally overwhelmed by those of ancestral demographic changes associated with paleo-climatic events, thanks to current advances in likelihood-based coalescent approaches to analyses of microsatellite data by building allelic genealogies^8^,^9^,^10^,^11^, which outperform traditional summary statistics in detecting recent or relatively weak demographic changes, we can now date and quantify the extent of expansions or contractions in recent population history^8^,^9^. Utilizing complex computational techniques, these novel approaches provide new genetic tools to evaluate the impact of recent human development and population expansion on the biodiversity of ecosystems^12^,^13^.

The Indo-Pacific humpback dolphin (*Sousa chinensis*), hereafter referred to as humpback dolphin, inhabits shallow (generally <25 m deep) coastal waters off southern China and Southeast Asia, and occurs primarily in highly productive estuarine habitats^14^,^15^,^16^. Most currently known populations of humpback dolphins are small in size, often <200 individuals^17^,^18^,^19^,^20^,^21^,^22^. Because of their restricted inshore distribution and habitat preferences that frequently bring them close to human populations and various human activities in the coastal zone, they are affected by a wide range of anthropogenic stressors^23^,^24^,^25^,^26^. In the Pearl River Delta (PRD) region, one of the largest estuarine areas within the species’ range, the population is thought to number some 2500 individuals^27^, making it apparently the world’s largest, and one of only two known populations of this species with a relatively large size^28^.

Lin *et al*.^29^ points out that the dropping sea level during glaciations would have led to a dramatic loss of coastal habitat for this species in southern China and Southeast Asia; and thus the humpback dolphin might have experienced bottleneck during the last glacial maximum (LGM, ~12 ka), similarly as found amongst other marine organisms inhabiting this region^30^,^31^. This hypothesis, however, which refers to the coastline evolution during the late Pleistocene^32^, requires further investigation as the regional coastline development during the Holocene has not received much attention^33^.

In the PRD plain, the livelihood of human populations has changed from hunting-gathering to farming around 3000–4000 years ago; and ever since the environment has been impacted by both climate (such as monsoons) and human activities^34^. Human-related impacts were not necessarily a dominant factor up until the Qin and Han Dynasties (221 B.C.−220 A.D.), the time of large human migration from central China to southern regions^34^,^35^. Migrants from the Yangtze River Basin have brought along advanced agricultural techniques and, within a period of Qin and Han dynasties (221 B.C.–220 A.D.), have led to a population explosion in the PRD region and large scale deforestation in the upper and middle reaches of the Pearl River^34^. Over the past 1000 years, the ever-increasing human population, development of rice cultivation, building of dikes and land reclamation have transformed the PRD into one of the most important economic centers of China. This process has been accompanied by local extinction of many terrestrial animals in the PRD plain^36^,^37^. At what degree these early anthropogenic impacts and human-induced environmental change have also affected coastal species remains poorly known, but impact on inshore marine mammals that depend on shallow-water inshore habitats cannot be ruled out.

Since the economic reform in mainland China in early 1980s, the PRD has changed rapidly into a highly urbanized industrial hub^38^. The economic “opening-up” has increased industrial and sewage pollution, overfishing, large-scale land reclamation, marine traffic and coastal constructions; which have resulted in a severe degradation of the marine ecosystem^39^. Thus it is not surprising that humpback dolphins in the NeiLingDing Sea, which is the most crowded and heavily used area in the PRD, have experienced a steady decline in the past decades^40^,^41^. These animals are one of the most anthropogenically impacted delphinids in the world^42^, yet their demographic history remains poorly known.

In the current study, we analyze the dynamics of the effective population size of humpback dolphins in the PRD. We evaluate the contribution of historic and recent bottleneck events to the current genetic pattern of the species and assess whether the causes were related to environmental and/or anthropogenic factors. The signals of a bottleneck were first evaluated using three traditional summary statistical methods, including two heterozygosity excess tests and the M-ratio. Second, the level and time of population decline, together with genetic parameters, were inferred using genealogy-based approaches developed by Beaumont^10^ and a similar approach refined by Storz *et al*.^43^, which assumes that microsatellites evolved under a stepwise mutation model (SMM model). A more recent approach developed by Leblois *et al*.^9^ follows a more realistic generalized stepwise model (GSM model) of microsatellite loci, and is applied here as it is expected to generate more accurate predictions of demographic parameters. Finally, we consider our results in a broader evolutionary context to postulate how demographic history and possible climatic and human-related forces may have driven the demographic change in the PRD humpback dolphins. We suggest that this hypothesis will broadly apply to this species across the Indo-Pacific region.

## Results

### Data delineation

**Figure 1:**
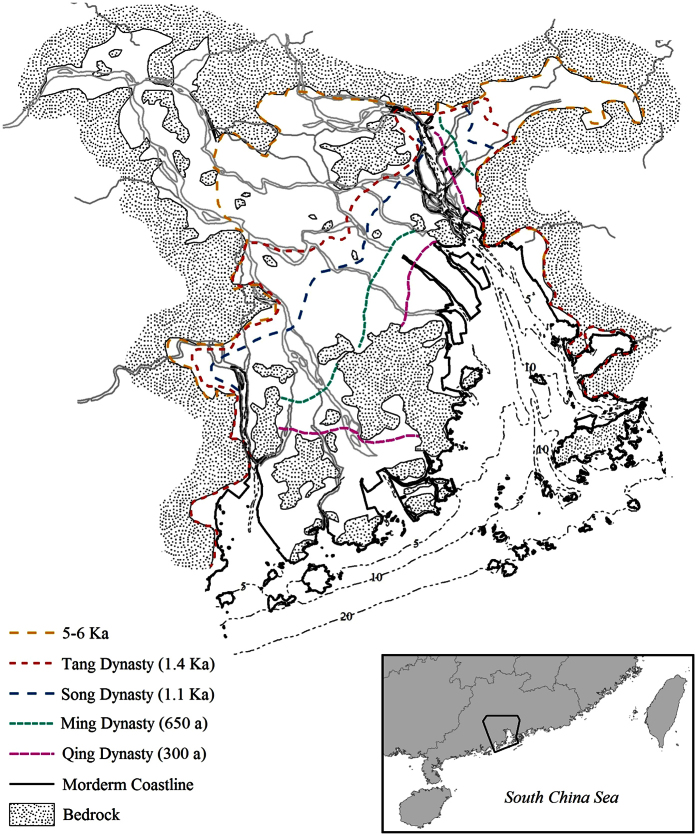
A map of the Pearl River Delta showing the present study area, along with the paleo coastlines reconstructed according to Zong *et al.*^34^. The dashed lines represent the −5 m, −10 m, and −20 m isobaths; the −20 m isobath is considered the limit of the humpback dolphins’ offshore distribution. The map was generated using ArcGIS 9.2. The coastline data is available on the web at http://www.naturalearthdata.com/downloads/ (Public Domain; date accessed: Jun 2011), and the hydrologic data is from http://www.mapcruzin.com/ (date accessed: Jun 2011) and licensed under CC-BY-SA 2.0 (https://creativecommons.org/licenses/by-sa/2.0/).

Of a total of 133 samples obtained from carcasses stranded between 2003 and 2014 in the PRD region (Fig. 1), 93 samples have generated unambiguous genetic results. Of the 15 microsatellite loci, the gene copies (2 × number of individuals) ranged from 88 to 136 (118 on average), with no sign of allelic dropout, null allele and stuttering. No evidence was detected for linkage-disequilibrium between loci except for one rare allele on SCA9 (2 out of 88) and one allele on SCA22 (1 out of 116, *p* = 0.021, Table 1). Since drift could also lead to a partial linkage-disequilibrium in a small population, all loci were retained for further analyses. The average allele number (*K*) and the expected heterogeneity averaged 3.53 (range: 2–11) and 0.385 (range: 0.149–0.750), respectively; this indicated a low level of gene diversity among the humpback dolphins in the PRD (Table 1). The number of clusters of individuals was estimated using a Bayesian clustering algorithm. As shown in Fig. S1, the sharp drop of LnP(D) after *K* > 1 strongly suggested that all of the samples used in the study came from a single population. Thus, the population contraction signal is unlikely to be due to the population structure.

### Homozygosity excess test and the M-ratio

If a population was constant in size, a microsatellite locus would show equal chance of either gene diversity excess or deficit as a result of mutation-drift equilibrium. During a bottleneck, however, the loss of allele numbers is faster than the rate of gene diversity. Thus, an observed excess of heterozygosity is generally interpreted as a sign of population reduction. Here, we used two summary statistical methods (Sign test and Wilcoxon test) to detect if the population contracted or expanded, based on the allelic frequency spectrum. Using the pure SMM model, neither test could reject the null hypothesis that the population was at mutation-drift equilibrium. Next, we ran the analyses using a more realistic TPM model, with 90% as the SMM model, which again showed no statistical support for a recent bottleneck. Although both tests showed significant diversity excess under the IAM model, this evolutionary model was not informative for microsatellite loci. Moreover, none of the tests remained significant after correction for a false discovery rate (Table S1). Similarly, the M-ratio also failed to detect the bottleneck. The observed value of M was 0.846835 under both scenarios (*θ* = 0.25 and *θ* = 2.5), which was close to but slightly larger than the simulated Mc (Mc = 0.844898 when *θ* = 0.25; Mc = 0.70651 when *θ* = 2.5).

### Results of the MSVAR analysis

**Figure 2:**
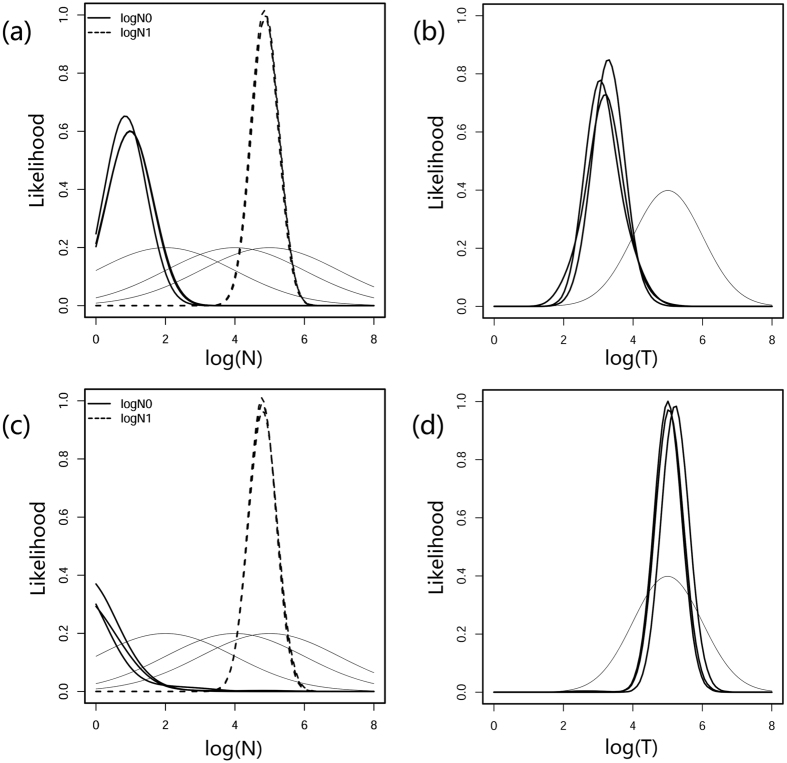
Inference of the demographic parameters using Storz-Beaumont’s method (MSVAR 1.3) with multiple replicates. For the effective population size under (**a**) an exponential model and (**c**) a linear model, the posterior density of the actual and pre-bottleneck sizes are denoted by thick and dotted lines, respectively. The posterior distribution of log(T) is shown in (**b,d**) under the demographic change model. The prior distributions of the parameters for independent replicates are presented with thin lines.

**Figure 3:**
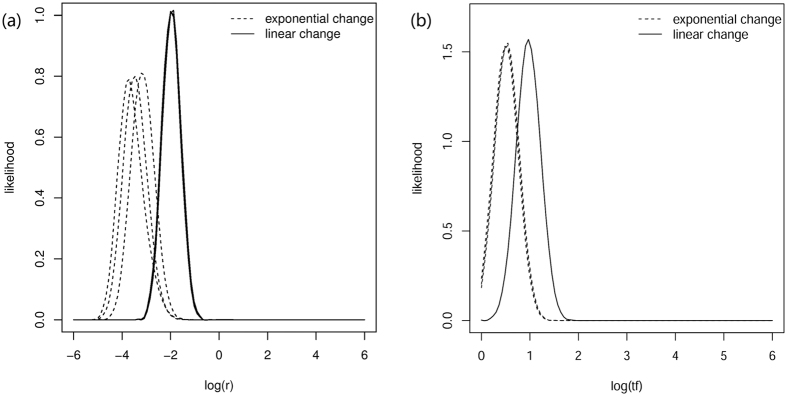
A population contraction was detected using the Beaumont’s approach (MSVAR 0.4) with multiple replicates. The posterior distribution of three independent replicates are plotted for (**a**) log(*r*) (*N*_0_*/N*_1_) and (**b**) log(*tf*) under an exponential model (dotted lines) and a linear model (solid lines). The negative log(*r*) indicates that the actual:ancestral effective population size ratio is smaller than 1 and, thus, indicates a population decline. The small value of log (*tf*) suggests that the decline occurred in recent history.

The summary statistical methods suffer from low detection power if a bottleneck occurred very recently or if the degree of contraction is relatively low. Therefore, we applied two Bayesian methods (Beaumont method and Storz-Beaumont method) to determine if a bottleneck occurred more recently and to gain further insights into the demographic history of the PRD humpback dolphins. The multivariate potential scale reduction factor indicated good convergence for most of the parameters (Table S2), with only exceptions for the log(*N*_0_) and log(*T*) which presented a thick tail of low estimates under the exponential model (Table S2, Fig. 2). As reaching convergence could be difficult with a recent and drastic bottleneck, we also considered any convergence after multiple runs that showed a stable distribution of the posterior distribution of parameters. As shown in Figs 2 and 3, all of the replicates using different random seeds and starting values showed a similar posterior distribution of demographic parameters. Thus, we combined the latter 50% of the iterations from different replicates for subsequent analyses.

The results of Beaumont method showed an unambiguously negative log(*r*) with a mode value of −3.87 (95% HPD: −3.98 to −3.14) under the exponential model and a mode value of −1.99 (95% HPD: −2.15 to −1.81) under the linear model. A point estimation of log(*tf*) was 0.53 (95% HPD: 0.41 to 0.63) under the exponential model, and 0.99 under the linear model (95% HPD: 0.89 to 1.00). The small value of log(*tf*) and negative log(*r*) indicated a population reduction within a recent time period (dozens or hundreds of generations) regardless of the demographic change models (Fig. 3).

The Storz-Beaumont method revealed a clear separation of log(*N*_1_) and log(*N*_0_; Fig. 2, Table 2), suggesting that the contemporary effective population size (*N*_0_) was substantially smaller than the ancestral size (*N*_1_). Log(*N*_1_) was estimated with high precision, while a flattened posterior distribution of log(*N*_0_) was observed. Log(*T*) was estimated to be higher under the linear model (mode value: 5.073, 95% HPD: 4.470–5.736) than under the exponential model (mode value: 3.332, 95% HPD: 2.582–4.057, Fig. 2). The *N*_1_/*N*_0_ ratio using the mode value was comparable to *r*. Using a generation time of 25 years (see Materials and Methods), the population reduction most likely began at 2.1 ka (95% HPD: 382–11403 years ago) under the exponential model, but was much earlier under the linear model (118.3 ka) with a wide range of 95% HPD interval (29.5 ka–544.5 ka).

### Result of MIGRAINE

Three runs of MIGRAINE with 2000 × 30 replicates are generally suggested to give reliable estimates for most of the demographic situations (program documentation available at http://raphael.leblois.free.fr/). To obtain sufficient data points for kriging, we had an initial simulation with three runs of 2000 × 2000 replicates. The lower bounds of 2Nμ and 2N_anc_μ were both set to 0.001, which corresponded to a population size as one individual with the mutation rate estimated by the MSVAR (2.5 × 10^−4^). The upper bound was set to 3 for 2Nμ, which was translated to a population size of 3000; and the value was doubled for 2N_anc_μ (Table S3). To further include the uncertainty of the mutation rate, we set the lower boundary of 2Nμ and 2N_anc_μ as 0.0005 in the second run, and the upper boundary of 2N_anc_μ as 10 in the final run of simulation (Table S3). Given that the effective population size is generally much smaller than the census population size, an upper boundary of 10 undoubtedly exceeds the possible range of this parameter. To ensure that the accuracy of the likelihood estimation is not affected by the number of replications, the second analysis was carried out with 8000 runs of simulation for each point (3 replicates × 3000 data points × 8000 runs per points), and the final simulation was repeated for six replicates (6 replicates × 3000 data points × 8000 runs per points). The results of last two simulations (Table 3) show no significant difference except for a slightly narrower 95% CI range of some parameters with more replicates, which suggested reliable inferences from these two analyses. The results of last run, which had the longest chain of simulation, were thought to be the most representative.

**Figure 4:**
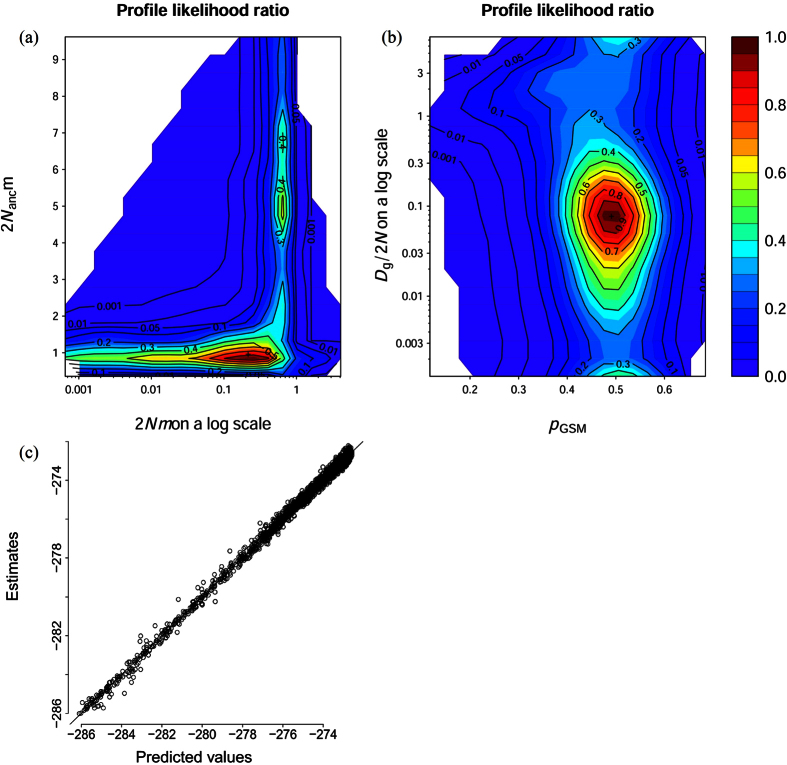
A two-dimensional profile likelihood ratio for (**a**) *θ* vs. *θ*_anc_, (**b**) Dg/2N vs. *p*GSM and (**c**) the diagnostic plot of kriging.

The likelihood ratio for ancestral/current *θ* (2N_anc_μ/2Nμ) and *p*GSM/Dg is shown in Fig. 4. Consistent with the results of the MSVAR analyses, the result of MIGRAINE indicated a strong and recent contraction of effective population size of the PRD humpback dolphins. Using a substitution rate of 2.5 × 10^−4^, the current and ancestral effective population size was estimated to be 264 and 893, respectively. There was a wide range for the inference of N_ratio_ (95% CI: 0.0000338–8.514) with a point estimation of 0.263, which could be translated into a 73.7% loss of the effective population size in the PRD within the latest decline. Given a generation time of 25 years and using the mode value of Dg/2N, the PRD humpback dolphin population has been in decline since 1.9 ka.

## Discussion

Our study provides a strong genetic evidence of a pronounced population contraction in the world’s largest population of Indo-Pacific humpback dolphins. The posterior distribution of log(*r*) (*r* = N_0_/N_1_) fell exclusively below 0 (Fig. 2), suggesting a substantially smaller current population size than the ancestral size. In our demographic model construction, equal probability was allowed for either population contraction or expansion, thus our finding appears robust. Even though we failed to detect any recent decline using the microsatellite allelic frequency (Table S1), this failure should not be interpreted as a sign of a stable population. Instead, it indicates that the decline of the PRD humpback dolphins occurred in relatively recent history rather than during the latest glaciation (~12 ka). This conclusion is based on the recognized difficulty of detecting deviations from mutation drift equilibrium for a recent decline (<10 generations) or relatively low contraction (N_0_/N_1_ > 0.1) using summary statistical methods^8^. The recent timing of the bottleneck is also supported by the incongruent precision of determining the ancestral and current effective population sizes. In case of ancestral bottleneck, most of the old mutations would have been lost and the dominating new mutations would have led to a more precise inference of the current population size^9^. In the present study, however, a skewed likelihood distribution was found for the current population size (Figs 2 and 4), which, together with the low value of *tf*, indicate that the population contraction does not predate the last glacial maximum or exceed hundreds of generations. Given that the environmental evolution of the PRD plain was influenced by climate change, human population growth and other human activities, accurate dating and quantification of the population contraction is critical to the understanding of evolutionary forces affecting this species.

**Figure 5:**
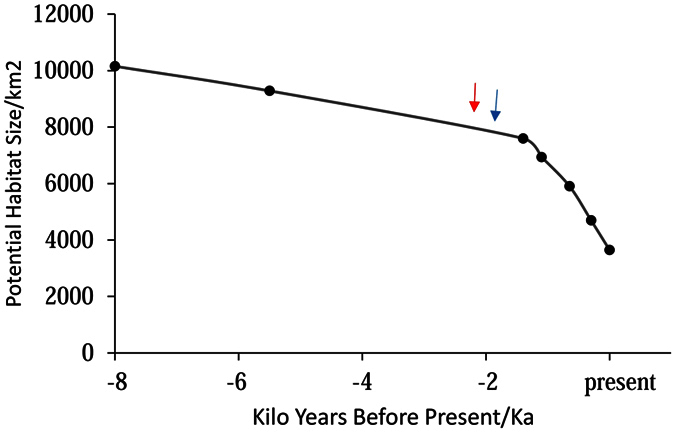
The reduction of potential habitat size for the Indo-Pacific humpback dolphins in the Pearl River Delta in the past 8,000 years. The blue and red arrows indicated the mode estiamte of the onset of population decline for the humpback dolphins by MIGRAINE and MSVAR, respectively.

As expected, simulations using different demographic models resulted in pronounced differences in the inference of the timing of the bottleneck (Fig. 2). Estimates under the linear model, which ranged from mid-Pleistocene to the end of late-Pleistocene (95% HPD: 29.5 ka–544.5 ka), provided little biological information about the possible time scale of population decline. An alternative estimate, using the exponential model, generated a narrower range of between 382 years ago and 11.4 ka, which is consistent with the history of estuarine evolution of the PRD during the Holocene (Fig. 5). This, along with the inference of effective population size indicates that exponential demographic model, not linear model, better describes our data.

After the end of last glacial maximum, the deltaic size reached its maximum at around 8 ka due to the rising sea level, and since then it has experienced a continuous marine regression^34^. At the beginning of marine regression, sedimentation was largely ascribed to climatic factor such as the formation of the East Asian Monsoon^44^. Human-environment interactions would have started with the first arrival of humans in the Middle Neolithic Age (some 5.2–6.5 ka)^35^. However, neither the climatic factor nor early human events, including the changing livelihood from hunting-gathering to farming around 3 to 4 ka^34^, could account for the humpback dolphin decline as the posterior support for the dates beyond 5.2 ka and 3 ka is just around 15.5% (against to a prior of 74.2%) and 35.0% (against to a prior of 84.1%), respectively (Fig. 2).

The role of climate in the deltaic evolution was likely surpassed by human activities around 2 ka due to two major events. Firstly, the PRD experienced a rapid growth of human population, owing to the large-scale migration from the central regions of China in the Qin Dynasty (221–207 B.C.)^35^. Secondly, advanced agricultural techniques, such as the slash-and-burn method, were introduced during this southward migration^34^,^35^. Increasing demand for food and farmland, and the slash-and-burn technique lead to large scale deforestation in southern China, which consequently increased the sediment influx and the sedimentation rate in the catchment area of the Pearl River. The soil erosion was further accelerated when rice cultivation shifted from the hills and terraces to the river valley^35^. As result of all these impacts, the sedimentation rate has tripled in the river outlet during the Song dynasty (960 A.D.–1279 A.D.) compared to earlier periods^34^. Given that the continental shelf was relatively stable since the last transgression^44^, the habitat size of humpback dolphins in the PRD has shrank steadily, with a linear decline, between 8 ka to 2 ka, but dropped sharply and exponentially in the last two thousand years with an increasing anthropogenic influence (Fig. 5). This turnover of human and climatic contribution to the deltaic evolution is consistent with the estimated onset of humpback dolphin population decline at 2.1 ka by MSVAR1.3 and 1.9 ka by MIGRAINE, which suggests that habitat loss caused by human development rather than climate was responsible for the population reduction of the PRD humpback dolphins in the recent history.

Generally, the substitution model of microsatellite loci may grant further insights into the demographic trajectory of a population. In our study, the *N*_0_ showed a 4–5 order of magnitude decline compared to *N*_1_ when SMM model was followed (Fig. 2). Even though such severe human-induced population collapse has been reported for terrestrial mammals, such as orangutans (*Pongo abelii* and *Pongo pygmaeus*) in Sumatra and Borneo^12^ and African elephants (*Loxodonta africana*) in South Africa^45^, it unlikely indicates the actual demographic history of the PRD humpback dolphins for two reasons. Firstly, 56.6% of the posterior distribution of *θ*_0_ suggested an effective population size <10 (Fig. 2), which, according to our field observations, is substantially less than the number of calves born in this area annually. Secondly, recent abundance estimates generated with line-transect techniques^27^ suggest that *ca.* 2,500 individuals inhabited the PRD in early 2010s, which would translate to an ancestral population of 25,000,000 individuals. Undoubtedly, this number exceeds the PRD’s capacity, even when the estuarine system reached its maximum size around 8 ka; thus it has little biological meaning.

Population fragmentation, which can also lead to severe reduction of effective population size even without significant loss in abundance, was not applied here as the recent fragmentation of humpback dolphin habitat on a larger regional scale^46^ has not yet left detectable genetic signatures^47^. Alternatively, it is possible that the extent of population reduction was exaggerated due to the violation of model assumption. For example, the Beaumont method and its extended version assumed a strict SMM model for microsatellite loci^10^,^11^. Violation of SMM model was generally negligible, but a considerable departure from the model assumptions (for example, *p*GSM as 49.1% [95% CI: 26–62%] as found in our dataset; Table 3) may lead to a large bias and decreases the accuracy and precision of estimated parameters, especially for *θ*_0_ and the current population size^8^. In this case, MIGRAINE enables more accurate estimates of demographic parameters by relaxing the assumption of a strict SMM model^9^. The point estimate of the present effective population size of 264 generated with MIGRAINE appears to be robust as the Ne/N ratio (11%) is well within a reasonable range reported for vertebrates^48^ and other cetaceans^49^. Furthermore, a population decline of approximately 73.7%, as suggested by the point estimate of MIGRAINE, is consistent with the reduction in habitat size in the past 2 ka (60.7%, Fig. 5). We failed, however, to improve the precision of inference despite a longer run and more replicates during the MIGRAINE analyses. The uncertainty of parameters might be due to insufficient information, given the low level of genetic variation among the humpback dolphins and the wide range of demographic scenarios to be tested. Moreover, the power of current analytical techniques is too low to detect the demographic event with the timing and magnitude that has been experienced by humpback dolphins in the PRD^10^,^11^.

The major objective of this present study was to evaluate the contribution of climate- and human-related factors on the demographic history of the world’s largest population of the Indo-Pacific humpback dolphin. Even though the contribution of climatic change could not be completely ruled out, given the wide 95% HPD range of the parameter; it received only 52.9% posterior support before 2 ka despite the much higher probability we included as a prior in the simulation (80.2%). However, as human-environment interactions are complex and have been influenced by both human population growth and the rapid development of agricultural techniques^35^, it was extremely difficult to single out specific individual components of anthropogenic impact as they are interconnected and not confined to the coastal region; e.g. land use practices and deforestation in the drainage area of an inland river will inevitably affect coastal ecosystem and its inhabitants. Over the past few hundred years, many large terrestrial mammals, birds and reptiles inhabiting the PRD plain have become extinct, including the Asian elephant (*Elephas maximus*), rhinoceros (*Rhinoceros spp.* and *Dicerorhinus sumatrensis*), crocodiles (*Crocodylus porosus* and *Tomistoma schlegelii*), green peafowl (*Pavo muticus*) and numerous other^37^,^38^,^50^. It was previously suggested that climate change was the primary cause of these extinctions in southern China. We, on the other hand, suggest that in the past few thousand years the climatic impact has been exacerbated and surpassed by human-caused environmental stress.

In conclusion, the present study indicates that habitat loss associated with human development is among the major historic causes of the decline of the PRD humpback dolphin population. As land use practices, deforestation, human migration and exploration have affected vast areas in a similar extent, coastal dolphins and large terrestrial mammals in southern China may share a similar demographic history. However, the extent of demographic decline of the humpback dolphin may differ between geographic populations due to differences in the sizes of river systems and urban areas, the extent of agricultural development and deforestation, and the sizes and shapes and carrying capacities of the receiving deltaic basins^34^. More recent forms of habitat degradation, such as large-scale land reclamation and coastal development, alteration and urbanization of coastlines, pollution, marine traffic, underwater noise, bycatch and resource overexploitation^26^ would not have left yet a detectable genetic signature and cannot be addressed at present with studies similar to ours. Their cumulative impacts, however, are likely far greater than what has been documented in our study. Given the obligatory inshore distribution of humpback dolphins and their dependence on restricted shallow-water habitats, in both PRD and elsewhere^51^,^52^,^53^, multifaceted protection of coastal habitats is instrumental in ensuring their continuous biological survival.

## Materials and Methods

The sampling area of the present study covers the whole PRD system, including the Neilingding Sea and Huangmao Sea (>250 km of coastline). The Neilingding Sea is the largest estuarine system of the PRD (2100 ha), connecting four of the eight branches of the Pearl River to the South China Sea. Seventy-seven samples were collected from carcasses stranded between 2003 and 2014 along the mainland P.R. China coast of the PRD, and 56 samples collected from carcasses stranded in Hong Kong were provided by the Ocean Park Conservation Foundation Hong Kong (OPCFHK) under the authorization of the Agriculture Fisheries and Conservation Department (Fig. 1). It has been suggested that the dolphin distribution shifts with the seasonal variation in freshwater discharge within the Neilingding Sea, towards outer reaches of the estuarine system during the wet season and back towards the river mouth in the dry season. Thus, the animals inside the Neilingding Sea are believed to belong to one demographic population.

### DNA preparation and microsatellite amplification

The entire genome was extracted from muscle (stored in ice) or skin (stored in formalin) using the phenol/chloroform method. Fifteen microsatellite loci (SCA9, SCA17, SCA22, SCA27, SCA37, SCA39, SCA54, SGATA25, SGATA30, SGATA42, SGATA45, Ttr11, Dde66, SCO11, and SCO28) were amplified using fluorescently labeled primers following the source papers^54^,^55^,^56^,^57^. The PCR products were sequenced using the commercial services of Invitrogen (Guangzhou, CN). The sizes of the alleles was determined using GeneMarker v2.2.0^58^. The presence of allelic dropout, null alleles and stuttering of microsatellite genotypes were tested with Micro-Checker 2.2.3^59^, and the linkage disequilibrium of pairwise loci was calculated using PopGene v1.3 http://www.ualberta.ca/ fyeh. Since the inference of demographic parameter could be biased with the presence of genetic structure, we used STRUCTURE 2.3.4 to reconstruct the hierarchical assignment of individuals following Bayesian clustering method^60^. The analyses were conducted under admixture model with the number of clusters (*K*) ranging from 1 to 10. Ten replicates were run for each *K*, with 10,000 burn-in period and then followed by 1,000,000 iterations.

### Analysis using BOTTLENECK and the M-ratio

In this study, a Sign test and Wilcoxon sign-rank test were performed under IAM, SMM and TPM models using BOTTLENECK v1.2.02^6^. Under the TPM model, the proportion of SMM was set at 90%, and the variance was set to 30 as default. The departure from equilibrium was evaluated using 1000 iterations. The M-ratio, which compares the number of alleles and the range of allele sizes, was also calculated to infer the demographic change using M_P_Val^61^. The average size of the multi-step mutations (Δg) and the proportion of one-step mutations (*ps*) were set as 3.5 and 0.9, respectively, as suggested by Garza and Williamson^61^. Two θ (θ = 4Ne*μ*) values were tested as 0.25 and 2.5, which assume an effective population size (Ne) of 250 and 2500, respectively, with a mutation rate (*μ*) of 0.00025, which was determined from the results of the MSVAR analysis. A critical threshold value (Mc) was determined using the 5% cut-off point of 10,000 simulated M-ratios to assess mutation-drift equilibrium using Critical_M. A significant deviation of the M-ratio from the null hypothesis was evident when the observed M-ratio fell below the Mc.

### Analysis using MSVAR

Beaumont^10^ suggests that summary statistical methods suffer from low detection power if a bottleneck occurred very recently or the degree of contraction was relatively low. Moreover, we could not date or quantify the bottleneck using these statistics. An alternative maximum-likelihood Bayesian method was developed^10^ to detect demographic change. This method assumes a simple model of an isolated population experiencing demographic change (either linear or exponential change) from *N*_1_ (effective population size before the change) to *N*_0_ (the present effective population size). Three parameters were calculated in MSVAR 0.4.2, including *r* (*N*_0_/*N*_1_), *tf* (Ta/N_0_), and *θ* (2*N*_0_μ). Under Beaumont’s method, the microsatellite was expected to evolve in the SMM model, thus allowing the program to trace back the genealogy of loci according to the prior distribution of each parameter following the Markov Chain Monte Carlo approach. The density of the posterior distribution of parameters was then estimated using the R package locfit (available at http://cran.r-project.org/web/packages/locfit/index.html), which referred to the point estimate and 95% CI of the parameters. This version of the program could quantify the degree of demographic change by estimating *r* in log transformation, with *r* > 1 indicating expansion and *r* < 1 representing demographic reduction. The date of the demographic change was also estimated and was scaled relative to *N*_0_.

This method was further improved by Storz-Beaumont^11^ in MSVAR 1.3, which allowed for the separate estimation of *N*_0_, *N*_1_, and (more importantly) the time (T) in years based on prior knowledge of the generation time of the species in question. Recent life table analyses suggest that the average life expectancy for the PRD humpback dolphins is 17 years (SD = 0.8) in the PRD^29^, which is considerably less than in eastern Taiwan Strait (21.3, SD = 3.8)^62^. These values are thought to be much lower than the natural life expectancy owing to the anthropogenic stress on both these populations; thus, the generation time was assumed to be equal to the average age of reproductively mature female humpback dolphins (age 25). In MSVAR 1.3, the prior distribution of each parameter was assumed to be log normal, while the mean value and standard deviation were further sampled from their hyperprior distribution.

When a 95% CI is considered, the range of N_0_ and N_1_ might overlap. Thus, analyses were run using both versions of MSVAR for a better description of the demographic history. For all these simulations, the prior distribution and variance of each parameter were set wide to avoid bias towards the hypothetical model. The number of iterations and lines of output were both initially set to 10,000 through a series of preliminary runs and then increased according to the results. The convergence of the simulations was tested with the Brooks, Gelman and Rubin Convergence Diagnostic (BGR Convergence Diagnostic) using the R package BOA (available at http://cran.r-project.org/web/packages/boa/index.html), which compared a sub-sample of simulations to the overall result. Estimates closer to one, with the 0.975 quantile ≤1.2, indicated good convergence^63^.

### Analysis using MIGRAINE

Nine out of the 15 microsatellite loci used in the present study are di-nucleotides, most of which do not evolve according to the SMM model. The violation of SMM may result in a false detection of the bottleneck signal. Thus, an alternative maximum-likelihood method (referred to as the Leblois *et al*. method hereafter) using a generalized stepwise mutation model (GSM) was adopted here. Leblois *et al*.^9^ improved a coalescent-based algorithm that samples all of the possible genealogies to generate a current genetic pattern. In the original algorithm, the resulting distribution of parameters under all of these possible genealogies relies on observed genetic data. Leblois *et al*.^9^ greatly improved the IS by allowing the population size to vary through time and by including a GSM model. Here, we used a panmictic population model with a variable population size (OnePopVarSize) in the program MIGRAINE (available at http://kimura.univ-montp2.fr/~rousset/MIGRAINE.html) to calculate five parameters: *p*GSM, 2Nμ, Dg/2N, 2N_anc_μ, and N_ratio_ (μ is the mutation rate per generation per locus; Dg is the time of the demographic change in the generation). The default boundaries of each parameter were chosen to allow for a wide search range. Up to 3000 parameter points were set for the initial run with 3 iterations, and each point was run 500 times. In a second simulation run, each point was increased to 2,000 runs, and the upper and/or lower boundary of each parameter was modified according to the result of the initial run. Then, in a third run, each point was increased to 20,000 runs, and the upper and/or lower boundary was modified if necessary. The procedure was repeated until the result remained stable with a low GOP value. Because the result of the MIGRAINE analysis is on a mutational scale, we transformed the three parameters (N, N_anc_, and T) into an actual number using μ as 2.5 × 10^−4^, and the generation was set to 25 years to further compare to the result of the MSVAR analysis.

## Additional Information

**How to cite this article**: Lin, W. *et al*. Increased human occupation and agricultural development accelerates the population contraction of an estuarine delphinid. *Sci. Rep.* **6**, 35713; doi: 10.1038/srep35713 (2016).

## Supplementary Material

Supplementary Information

## Figures and Tables

**Table 1:** Molecular diversity and test results of null alleles and linkage disequilibrium (LD).

Locus	n	N	*H*_o_	*H*_e_	Null allele	LD
SCA9	88	11	0.750	0.827	NA	P
SCA22	116	8	0.707	0.773	NA	P
SCA27	118	3	0.407	0.433	NA	NA
SCA37	104	3	0.212	0.193	NA	NA
SCA54	92	3	0.152	0.145	NA	NA
SGATA30	114	2	0.228	0.256	NA	NA
SGATA42	94	2	0.149	0.209	NA	NA
SGATA45	94	2	0.170	0.157	NA	NA
SCA17	114	3	0.526	0.546	NA	NA
SCA39	108	3	0.278	0.320	NA	NA
SGATA25	98	2	0.306	0.316	NA	NA
Ttr11	110	3	0.600	0.614	NA	NA
Dde66	112	3	0.589	0.546	NA	NA
SCO11	94	2	0.383	0.339	NA	NA
SCO28	118	3	0.322	0.320	NA	NA
Mean	105	3.53	0.385	0.400		
S.D.		2.53	0.203	0.217		

N, the allele number; n, the samples size; NA, no significant departure from the null hypothesis; P, suggests partial linkage-disequilibrium between loci.

**Table 2:** The mode and 95% HPD of demographic parameters estimated by the Storz-Beaumont method (MSVAR 1.3).

	Linear model			Exponential model		
	mode	lower bound	upper bound	mode	lower bound	upper bound
log(*N*_1_)	4.801	4.190	5.474	4.879	4.262	5.459
log(*N*_0_)	−0.357	−2.434	1.035	0.922	0.149	1.718
log(μ)	−3.600	−4.085	−3.129	−3.608	−4.087	−3.129
log(T)	5.073	4.470	5.736	3.332	2.582	4.057

**Table 3:** Point estimates and 95% confidence intervals (in parentheses) of the demographic parameters estimated with MIGRAINE analyses.

	*p*GSM	2Nμ	Dg/2N	2N_anc_μ	N_ratio_
1	0.491 (0.284–0.624)	0.215 (NA–1.372)	0.0766 (NA–NA)	0.952 (0.138–6.02)	0.226 (0.000453–2.827)
2	0.491 (0.268–0.621)	0.264 (0.000032–3.329)	0.0715 (NA–35.83)	0.893 (0.553–6.293)	0.295 (NA–4.279)
