# Supplementary material for: Increased human occupation and agricultural development accelerates the population contraction of an estuarine delphinid
Source: Sci Rep. 2016 Oct 19;6:35713. doi: 10.1038/srep35713 (PMC5069629; doi:10.1038/srep35713)
Supplement: Supplementary Information [file srep35713-s1.pdf]

Increased human occupation and agricultural development accelerates the population contraction of an estuarine delphinid

Wenzhi Lin[1, 2], Leszek Karczmarski[2, *], Jia Xia[1], Xiyang Zhang[1], Xinjian Yu[1], Yuping Wu[1, *]

1 South China Sea Bio-Resource Exploitation and Utilization Collaborative Innovation Center; Zhuhai Key Laboratory of Marine Bioresources and Environment, Guangdong Provincial Key Laboratory of Marine Resources and Coastal Engineering, School of Marine Sciences, Sun Yat-Sen University, Guangzhou 510275, PR China;

2 The Swire Institute of Marine Science and School of Biological Sciences, The University of Hong Kong, Cape d'Aguilar, Shek O, Hong Kong.

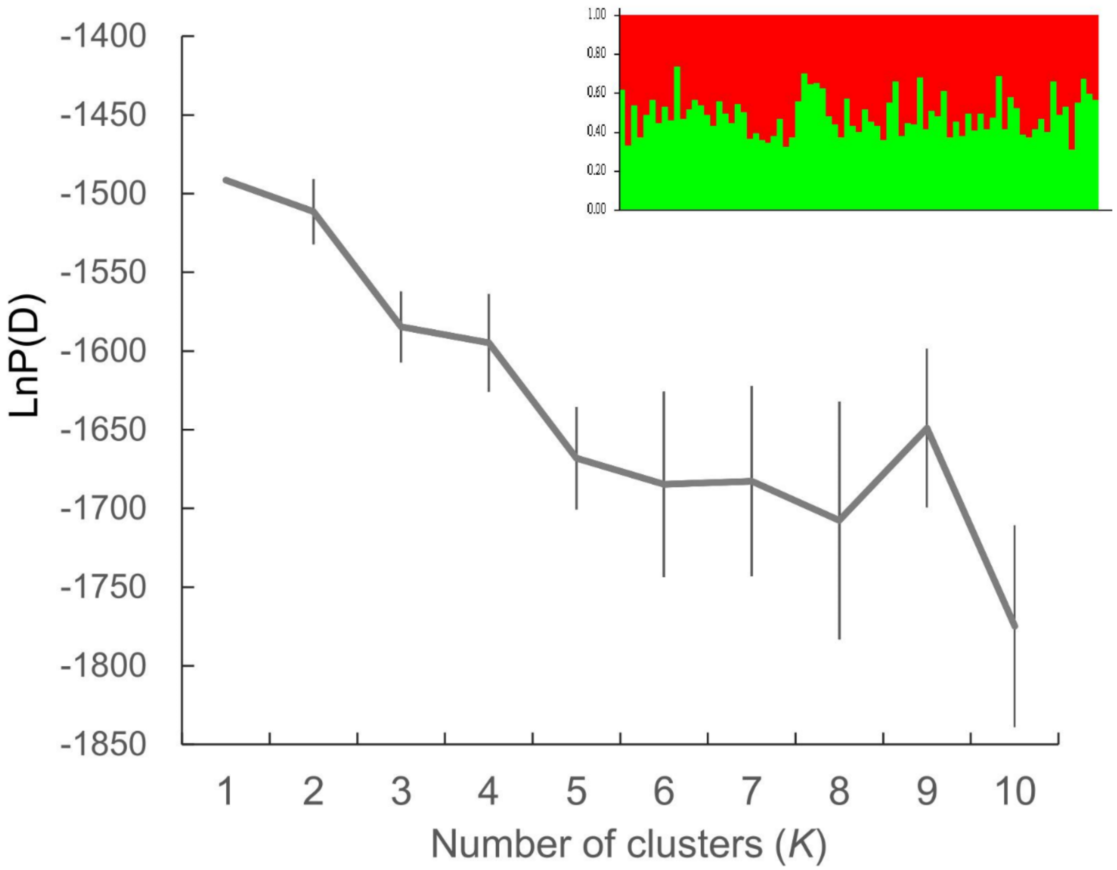

Fig S1. Bayesian clustering of individual using program STRUCTURE with the number of clusters (K) ranging from 1 to 10. The highest LnP(D) was found when *K*=1, which steadily dropped without overlapping variance when 3≥*K*≥1. The panels on the top right shows that the likelihood of individual coming from each putative population was very close to each other when *K*=2, which provides little biological meaning. Thus *K*=1 is the most biologically meaningful structuring of the current genetic dataset.

Table S1 Calculation of the heterozygosity excess under different mutation models of *Sousa chinensis* in the PRE to test for a recent bottleneck event.

	Sign Test Hee	He	uncorrected *p*	corrected *p*	Wilcoxon Test	uncorrected *p*	corrected *p*
I.A.M	7.25	11	0.04413	0.01111	P(one tail for H excess)	0.05063	0.01667
					P(two tails for H excess and deficiency)	0.04126	0.00556
S.M.M	8.29	6	0.17325	0.02222	P(one tail for H excess)	0.82043	0.05000
					P(two tails for H excess and deficiency)	0.38940	0.03333
T.P.M	8.21	6	0.18425	0.02778	P(one tail for H excess)	0.73776	0.04444
					P(two tails for H excess and deficiency)	0.56140	0.03889

Hee, expected Heterozygosity excess under mutation-drift equilibrium; He, observed Heterozygosity excess; uncorrected *p*, default *p*-value of 0.05; corrected *p*, the *p*-value corrected using false discovery rate statistics. A significant deviation from the null hypothesis is defined as when the uncorrected *p* is smaller than the corrected *p*.

Table S2 Brooks, Gelman and Rubin convergence diagnostic results of simulations using the Beaumont and Storz-Beaumont methods

methods	Parameters	Exponential model		Linear model	
		Estimate	0.975	Estimate	0.975
Storz-Beaumont method	log(μ)	1.000619	1.002289	1.003029	1.010603
	$\log(N_0)$	1.152991	1.448031	1.041185	1.10592
	$\log(N_1)$	1.000985	1.003521	1.000112	1.000385
	log(T)	1.154144	1.450872	1.010401	1.034126
Beaumont method	log(*r*)	1.088419	1.311555	1.000029	1.000040
	log(*tf*)	1.160790	1.560290	1.000010	1.000082

Table S3 Setting for MIGRAINE analysis. Column 1 and 2 represent the number of points per simulation and the number of runs per point. The population is expected to experience a continuous decline until the present time; thus, T_{change} is set to zero and not shown here. According to the results of the MSVAR analysis, more informative prior ranges were set for 2Nμ and $2N_{anc}\mu$.

PointNum	NrunsperPoint	Nruns	pGSM		2Nμ		Dchange		$2N_{anc}\mu$	
			Lower Bound	Upper Bound	Lower Bound	Upper Bound	Lower Bound	Upper Bound	Lower Bound	Upper Bound
2000	2000	3	0.1	1	0.001	3	0.001	10	0.001	6
3000	8000	3	0.1	1	0.0005	3	0.001	10	0.0005	6
3000	8000	6	0.1	0.7	0.0005	10	0.001	10	0.0005	10
